# Updates on the Role of ABSCISIC ACID INSENSITIVE 5 (ABI5) and ABSCISIC ACID-RESPONSIVE ELEMENT BINDING FACTORs (ABFs) in ABA Signaling in Different Developmental Stages in Plants

**DOI:** 10.3390/cells10081996

**Published:** 2021-08-05

**Authors:** Anna Collin, Agata Daszkowska-Golec, Iwona Szarejko

**Affiliations:** Institute of Biology, Biotechnology and Environmental Protection, Faculty of Natural Sciences, University of Silesia in Katowice, ul. Jagiellońska 28, 40-032 Katowice, Poland; anna.skubacz@us.edu.pl (A.C.); iwona.szarejko@us.edu.pl (I.S.)

**Keywords:** ABI5, ABF, AREB, abiotic stress response, abscisic acid, phytohormone crosstalk

## Abstract

The core abscisic acid (ABA) signaling pathway consists of receptors, phosphatases, kinases and transcription factors, among them ABA INSENSITIVE 5 (ABI5) and ABRE BINDING FACTORs/ABRE-BINDING PROTEINs (ABFs/AREBs), which belong to the BASIC LEUCINE ZIPPER (bZIP) family and control expression of stress-responsive genes. ABI5 is mostly active in seeds and prevents germination and post-germinative growth under unfavorable conditions. The activity of ABI5 is controlled at transcriptional and protein levels, depending on numerous regulators, including components of other phytohormonal pathways. ABFs/AREBs act redundantly in regulating genes that control physiological processes in response to stress during vegetative growth. In this review, we focus on recent reports regarding ABI5 and ABFs/AREBs functions during abiotic stress responses, which seem to be partially overlapping and not restricted to one developmental stage in Arabidopsis and other species. Moreover, we point out that ABI5 and ABFs/AREBs play a crucial role in the core ABA pathway’s feedback regulation. In this review, we also discuss increased stress tolerance of transgenic plants overexpressing genes encoding ABA-dependent bZIPs. Taken together, we show that ABI5 and ABFs/AREBs are crucial ABA-dependent transcription factors regulating processes essential for plant adaptation to stress at different developmental stages.

## 1. Insight into the Core ABA Signaling and ABA-Dependent bZIPs Function

Each year abiotic stresses, including drought and salinity, reduce crop yield, causing economic problems and a severe threat to food safety. Thus, it is crucial to understand plant adaptation mechanisms to unfavorable environmental conditions with aim to develop stress-tolerant cultivars [1,2]. Abscisic acid (ABA) is a significant phytohormone regulating plant responses to various abiotic stresses [3,4]. In response to stress, ABA is synthesized in plant cells triggering activation of the ABA signaling pathway. The core ABA signaling consists of ABA receptors (PYRABACTIN RESISTANCE PROTEINS/PYR-LIKE PROTEINS/REGULATORY COMPONENTS OF ABA RECEPTOR; PYR/PYL/RCAR), phosphatases (PHOSPHATASE 2Cs; PP2Cs), kinases (SNF1-RELATED PROTEIN KINASE 2; SnRK2s), and transcription factors belonging to large BASIC LEUCINE ZIPPER (bZIP) family. After perceiving the primary stress signal, ABA forms a complex with PYR/PYL/RCAR receptors and PP2C phosphatases. It prevents PP2Cs from dephosphorylating SnRK2s and releases a SnRK2s phosphorylation activity. SnRK2 kinases phosphorylate and activate bZIP transcription factors, such as ABA INSENSITIVE 5 (ABI5) and ABRE BINDING FACTORs/ABRE-BINDING PROTEINs (ABFs/AREBs) [5,6,7]. However, it has to be underlined that ABA-dependent bZIPs are also phosphorylated by other kinases [8,9,10]. Recently, it was found that RAF-LIKE KINASE 10 (RAF10) and CALCIUM-DEPENDENT PROTEIN KINASE 6 (CPK6) interact with ABI5 and ABFs/AREBs and phosphorylate them [11,12]. Moreover, RIBOSOMAL S6 KINASE2 (S6K2), kinase active in TARGET OF RAPAMYCIN (TOR) signaling, was also shown to bind with ABI5 what in turn stimulates ABA response and drought tolerance [13]. Phosphorylated ABI5 and ABFs/AREBs recognize ABA RESPONSIVE ELEMENTs (ABRE cis-elements) containing (C/T)ACGTGGC motif and G-box coupling elements (GCEs) with ACGT/C core sequence, present in the promoters of stress-responsive genes. These bZIP factors activate or repress their expression and trigger plant adaptation to stress [5,14,15].

In our previous review [10], we described the function of AtABI5 and its homologs in the acquisition of stress tolerance in plants. However, recently numerous new data emerged about ABI5 and ABFs/AREBs function under stress. This review presents new evidence on ABI5 and ABFs/AREBs role in plant adaptation to stress and describes a tight control of their activity by other stress regulators. We also focus on ABI5 and ABFs/AREBs actions in the feedback regulation of ABA pathway and the possibility of utilizing ABA-dependent bZIPs in developing stress-tolerant cultivars.

## 2. New Evidence of ABI5 Regulatory Role during Seed Germination

**Figure 1:**
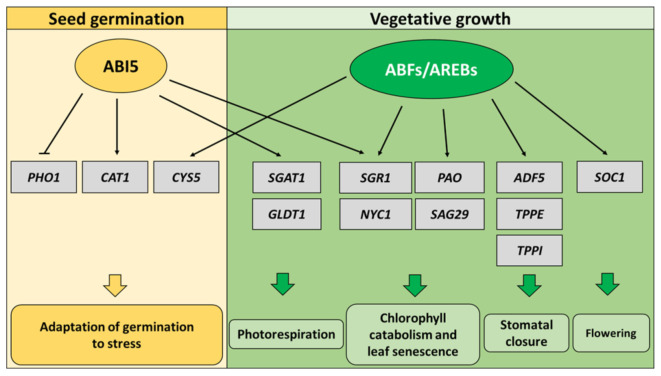
Partially overlapping function of ABA INSENSITIVE 5 (ABI5) and ABRE BINDING FACTORs/ABRE-BINDING PROTEINs (ABFs/AREBs). ABI5 regulates seed germination accordingly to surrounding environmental conditions. ABI5 promotes *CATALASE 1* (*CAT1*) and represses *PHOSPHATE1* (*PHO1*), responsible for reactive oxygen species (ROS) scavenging and phosphate transfer, respectively, during seed germination, while ABFs/AREBs ensure stress adaptation in vegetative tissues. Genes associated with chlorophyll catabolism (*STAY-GREEN 1*—*SGR1*, *NON-YELLOW COLORING 1*—*NYC1*), leaf senescence (*PHEOPHORBIDE A OXYGENASE*—*PAO*, *SENESCENCE-ASSOCIATED GENE 29*—*SAG29*), stomatal closure (*ACTIN-DEPOLYMERIZING FACTOR 5*—*ADF5*, *TREHALOSE-6-PHOSPHATE PHOSPHATASE E*—*TPPE*, *TPPI*) and flowering time (*SUPPRESSOR OF OVEREXPRESSION OF CO 1*—*SOC1*) are the direct targets of ABFs/AREBs. ABI5 is also able to promote expression of chlorophyll catabolism (*SGR1*, *NYC1*) and photorespiration (*SERINE:GLYOXYLATE AMINOTRANSFERASE 1*—*SGAT1*, *GDC T-PROTEIN*—*GLDT1*) genes in vegetative tissues, whereas ABFs/AREBs activate expression of *CYSTEINE PROTEINASE INHIBITOR 5* (*CYS5*) in seeds.

In *Arabidopsis thaliana*, ABA-induced activation of ABI5 inhibits germination under unfavorable environmental conditions. ABI5 is responsible for the regulation of expression of stress-responsive genes, e.g., *EARLY METHIONINE-LABELED 1* (*EM1*) and *EM6* encoding LATE EMBRYOGENESIS ABUNDANT (LEA) proteins [10,16,17]. Recently, ABI5 was shown to repress the expression of *PHOSPHATE1* (*PHO1*), a gene involved in phosphate (Pi) transfer from cotyledons to radicles which promotes germination. Therefore, one of the mechanisms of ABI5-dependent germination inhibition is repression of Pi transfer [18]. Furthermore, ABI5 can directly activate *CATALASE 1* (*CAT1*), encoding a catalase responsible for scavenging H_2_O_2_, the main reactive oxygen species (ROS). It indicates that ABI5 is also involved in maintaining ROS homeostasis during seed germination [19] (Figure 1).

Multiple transcription factors regulate *ABI5* expression during seed germination in Arabidopsis [10,20,21,22]. Recently, the new regulator of *ABI5* in seeds was identified. AGAMOUS-LIKE 21 (AGL21) belongs to MCM1/AGAMOUS/DEFICIENS/SRF (MADS) box group of transcription factors. It was shown that AGL21 activates the expression of *ABI5* in ABA-treated seeds in Arabidopsis [23]. 

**Figure 2:**
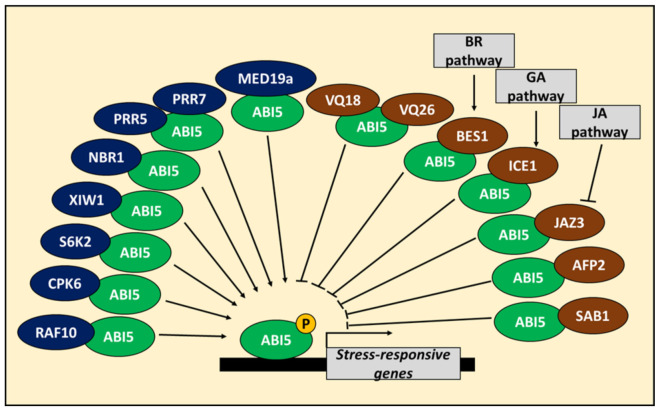
Positive and negative regulators of ABA INSENSITIVE 5 (ABI5) protein stability and/or function. ABI5 stability and activity is under regulation of multiple proteins. ABI5 stability and function is promoted by interaction with kinases CALCIUM-DEPENDENT PROTEIN KINASE 6 (CPK6), RAF-LIKE KINASE 10 (RAF10) and RIBOSOMAL S6 KINASE2 (S6K2), shuttle protein XPO1-INTERACTING WD40 PROTEIN 1 (XIW1), circadian clock regulators PSEUDO-RESPONSE REGULATOR 5 (PRR5) and PRR7 and mediator subunit MEDIATOR COMPLEX SUBUNIT 19a (MED19a). ABI5 is also stabilized by interaction with NEIGHBOUR OF BREAST CANCER 1 (NBR1). On the other side, ABI5 stability and/or function is negatively affected by VQ18, VQ26, BRASSINOSTEROID INSENSITIVE 1 (BRI1)-EMS-SUPPRESSOR 1 (BES1), INDUCER OF CBF EXPRESSION1 (ICE1), JASMONATE-ZIM DOMAIN PROTEIN 3 (JAZ3), ABI5 BINDING PROTEIN 2 (AFP2) and SENSITIVE TO ABA 1 (SAB1). BES1, ICE1 and JAZ3 are also involved in brassinosteroid (BR), gibberellic acid (GA) and jasmonic acid (JA) signaling, respectively. P—phosphate group.

In Arabidopsis, ABI5 is also regulated at the protein level. It interacts with numerous proteins, modulating its stability or activity as a transcription factor [10,24,25,26,27]. Recently, XPO1-INTERACTING WD40 PROTEIN 1 (XIW1), a member of WD40-repeat protein (WD40) family, was found to be crucial for ABI5 stability during seed germination. XIW1 shuttles between nucleus and cytoplasm dependently on environmental conditions. ABA promotes nuclear localization of XIW1. Colocalization of XIW1 and ABI5 in the nucleus leads to their physical interaction and, thus, protects ABI5 from proteasomal degradation, which enables ABA-mediated responses in seeds [28] (Figure 2). Furthermore, the interaction between ABI5 and autophagy cargo receptor, NEIGHBOUR OF BREAST CANCER 1 (NBR1), may enhance ABI5 stability and ABA response at the germination stage [29,30]. Recently, Yang et al. [31] showed that ABI5 activity is regulated by circadian clock regulators, PSEUDO-RESPONSE REGULATOR5 (PRR5) and PRR7. Both proteins interact physically with ABI5 to stimulate ABA response in seeds and to inhibit germination according to day/night cycle (Figure 2). Moreover, Pan et al. [32] discovered that ABA-induced members of the newly identified VQ motif-containing protein (VQ) family, VQ18 and VQ26, interact physically with ABI5 and block its function (Figure 2). VQ18 and VQ26 were proposed to be a part of the fine-tuning mechanism in the frame of ABA signaling during seed germination. Furthermore, ABI5 BINDING PROTEIN 2 (AFP2) was found to interact with ABI5 and inhibits its transactivation of *SOMNUS* (*SOM*), encoding a negative regulator of GA accumulation and seed germination under high temperature [33] (Figure 2). It is noteworthy that another AFP protein, AFP1, was shown to promote ABI5 degradation [25]. 

## 3. Involvement of ABI5 in Phytohormonal Crosstalk at the Seed Germination Stage

ABI5 can act as a hub in phytohormonal crosstalk in Arabidopsis [10,27,34,35,36,37]. The last reports confirm this hypothesis and indicate that at the seed germination stage ABI5 plays an essential role in brassinosteroid (BR), gibberellic acid (GA), cytokinin (CK) and jasmonic acid (JA) signaling through interaction with components of these pathways [38,39,40,41]. Zhao et al. [38] showed the interaction between ABI5 and BRASSINOSTEROID INSENSITIVE 1 (BRI1)-EMS-SUPPRESSOR 1 (BES1), a BR-dependent transcription factor and negative regulator of ABA signaling. BES1 inhibits ABI5 activity and thus promotes seed germination (Figure 2). Interestingly, binding of BES1 to ABI5 prevents the ABI5 interaction with ABI3, the enhancer of ABI5 transactivation function [38]. Similarly, INDUCER OF CBF EXPRESSION1 (ICE1) transcription factor binds and represses ABI5 transactivation function in seeds. This interaction is promoted by GA, but inhibited by DELLA proteins, negative components of GA signaling, which also bind to ICE1 and therefore restore ABA signaling and ABI5 function [39]. Furthermore, CK-dependent regulators ARABIDOPSIS RESPONSE REGULATOR 4 (ARR4), ARR5 and ARR6 inhibit *ABI5* expression at germination stage [42], whereas other components of CK signaling, ARABIDOPSIS HISTIDINE KINASE 4 (AHK4), ARABIDOPSIS HISTIDINE PHOSPHOTRANSFER PROTEIN 2 (AHP2), AHP3, AHP5 and ARR12 trigger ABI5 protein degradation during cotyledon greening [36]. Moreover, JASMONATE-ZIM DOMAIN PROTEIN 3 (JAZ3), a negative regulator of JA response, interacts with ABI5 and reduces its activity as transcription factor in germinating seeds (Figure 2). However, ABA treatment promotes JAZ3 degradation and JA biosynthesis in the ABI5-dependent way [40]. Recently, it was shown that JAZ proteins repress *ABI3* and *ABI5* expression, while JA stimulates ABA response through regulation of *ABI3* and *ABI5* during seed germination inhibition [41].

## 4. The Function and Regulation of ABI5 during Seedling Development

ABI5 plays the essential role in repression of seedling growth under osmotic, salt and cold stress in Arabidopsis [10,43,44]. It was recently found that ABI5 may also be essential for seedling growth tolerance to aluminum (Al) through expression regulation of genes related to cell wall modification and osmoregulation [45]. New evidence also emerged about *ABI5* transcriptional regulation in Arabidopsis seedlings. It was shown that NAM/ATAF1/2/CUC2 (NAC) transcription factor, ANAC060, represses *ABI5* activity in seedlings in response to glucose. Interesting, it can be a part of negative feedback regulation between ABI5 and another ABA-dependent and glucose-related transcription factor, ABI4, which promotes *ANAC060* expression [46]. Previously, B-BOX DOMAIN PROTEIN 21 (BBX21) was shown to inhibit the *ABI5* expression under light [47]. Kang et al. [48] revealed that BBX21 represses *ABI5* by recruiting its promoter chromatin modifier, HYPERSENSITIVE TO RED AND BLUE 2/PICKLE (HRB2/PKL). Decreased *ABI5* expression results in maintenance of stomatal aperture at the level that is necessary for gas exchange, however, the precise mechanism is unknown. Therefore, these data indirectly indicate that ABI5 is responsible for the regulation of stomata movement in seedlings [48]. In the dark, *ABI5* expression is activated by PHYTOCHROME-INTERACTING FACTORs (PIF): PIF1, PIF3, PIF4 and PIF5 that are the negative regulators of photomorphogenesis [49]. Interestingly, previously ABI5 was shown to interact with PIF1 to strengthen its function as a transcription factor [50]. WRKY18, WRKY40 and WRKY60 are negative regulators of *ABI5* transcription in seedlings [10,51]. Recently, WRKY40 was found to recruit a histone 3 lysine 4 (H3K4) demethylase JUMONJI DOMAIN-CONTAINING PROTEIN 17 (JMJ17) to chromatin of *ABI5* gene. JMJ17 removes marks of transcriptionally active chromatin (H3K4me3) from *ABI5* and thus also inhibits *ABI5* expression at epigenetic level in seedlings under non-stressed conditions [52].

Other proteins also modulate ABI5 activity during seedling development in Arabidopsis. Similar to seeds, ABI5 stability is regulated by XIW1 and NBR1 at the seedling stage [28,29]. Furthermore, MEDIATOR COMPLEX SUBUNIT 19a (MED19a) physically interacts with ABI5 during ABA-dependent inhibition of root growth and cotyledon greening (Figure 2). It was shown that MED19a strengthens ABI5 binding to the promoters of *EM1* and *EM6* [53]. It has to be underlined that ABI5 is negatively regulated by another MEDIATOR subunit, MED25 [10,35]. The next identified ABI5 interactor, SENSITIVE TO ABA 1 (SAB1), in many ways inhibits ABI5 activity during early seedling growth. SAB1 belongs to REGULATOR OF CHROMATIN CONDENSATION 1 (RCC1) family and it binds to ABI5 serine at 145 position, which serves as a target of SnRK2s-mediated phosphorylation. The SAB1 binding causes reduction of ABI5 phosphorylation status and leads to its degradation (Figure 2). SAB1 also binds to *ABI5* promoter and inhibits ABI5 to auto-activate its expression. Moreover, SAB1 increases the level of histone H3K27me2, the epigenetic mark of expression repression, in the *ABI5* promoter [54]. Together, all these findings show that ABI5 activity undergoes complex and strict regulation, which shows that ABI5 plays an essential role in ABA signaling during early developmental stages. It has to be pointed out that ABI5 can interact with other transcription factors to regulate their activity. ABI5 is involved in anthocyanin accumulation in seedlings [44]. Recently, it was shown that in apple (*Malus domestica*), homolog of AtABI5, MdABI5, binds with basic helix-loop-helix 3 (MdbHLH3) which in turn enhances expression of its target genes, *DIHYDROFLAVONOL 4-REDUCTASE* (*MdDFR*) and *UDP FLAVONOID GLUCOSYL TRANSFERASE* (*MdUF3GT*), involved in anthocyanin biosynthesis. Moreover, MdABI5 strengthens interaction between MdbHLH3 and MdMYB1, another transcription factor involved in anthocyanin biosynthesis. MdABI5 can also promote directly expression of *MdbHLH3* [55].

## 5. Redundant Function of ABFs/AREBs in the Regulation of Plant Stress Responses in Vegetative Tissues

ABFs/AREBs: ABF1, ABF2/AREB1, ABF4/AREB2, and ABF3 regulate plant response to abiotic stresses, such as drought, salt, heat, oxidative stress, and cold, mostly in vegetative tissues of Arabidopsis [56,57,58,59]. ABFs/AREBs ensure the adaptation to unfavorable environmental conditions via promoting expression of *LEA* genes, such as *RESPONSIVE TO DESSCICATION 29B* (*RD29B*), *RESPONSIVE TO ABA 18* (*RAB18*), *COLD-RESPONSIVE 6.6* (*COR6.6/KIN2*), and regulatory genes, such as *DEHYDRATION-RESPONSIVE ELEMENT BINDING PROTEIN 2A* (*DREB2A*) [14,57,60]. However, ABFs/AREBs role in ABA-mediated adaptation to stress is highly redundant. Quadruple mutant *abf2/areb1 abf4/areb2 abf3 abf1* showed a significantly lower survival rate after drought treatment than single *abf/areb* mutants. Similar observations were made for the primary root growth of *abf/areb* mutants in the presence of ABA [14,61]. Moreover, ABFs/AREBs can interact with each other and function together to regulate expression of target genes [8,14]. On the other side, ABF2/AREB1, ABF4/AREB2, and ABF3 regulate the expression of *RD29B* or *RAB18* in a slightly different way, which indicates the partially independent role of each *ABF/AREB* during adaptation to stress [14].

*ABFs/AREBs* regulate redundantly also stomatal closure in Arabidopsis. *ACTIN-DEPOLYMERIZING FACTOR 5* (*ADF5*) encodes a protein responsible for actin cytoskeleton remodeling during stomatal closure in response to ABA. ABF1, ABF2/AREB1, ABF4/AREB2 and ABF3 bind to *ADF5* promoter, activate its expression, promote stomatal closure and ensure adaptation to drought [15]. It was recently shown that ABFs/AREBs-mediated stomatal closure depends on accumulation of disaccharide trehalose in seedlings. ABF1, ABF2/AREB1 and ABF4/AREB2 directly bind to the promoter of *TREHALOSE-6-PHOSPHATE PHOSPHATASE I* (*TPPI*) and promote its expression. *TPPI* gene encodes the trehalose biosynthesis enzyme [62]. Additionally, ABF2/AREB1 directly activates another gene from *TPP* family, *TPPE* [63] (Figure 1). Strikingly, *TPPI* is involved in promoting primary root growth, whereas *TPPE* takes part in ABA-dependent inhibition of root growth [62,63]. Homologs of AtABF/AREB in other dicot species are also involved in stomata regulation. In cotton (*Gossypium hirsutum*), *GhABF2D* promotes stomatal closure and thus abiotic stress tolerance [64]. Moreover, carrot (*Daucus carota*) DcABF3 activates expression of genes involved in stomata development, *SPEECHLESS* (*SPCH*), *FAMA* (*FMA*) and *MUTE*, which in turn increases number of stomata [65].

In vegetative tissues of Arabidopsis, ABFs/AREBs are regulated at transcriptional, post-transcriptional and protein levels. Expression of *ABFs/AREBs* is under control of other stress-responsive transcription factors, NACs. NAC016 and NAC-LIKE, ACTIVATED BY AP3/PI (NAP) were shown to repress *ABF2/AREB1* under drought [66]. Additionally, NAP negatively regulates also salt stress response by inhibiting *ABF2/AREB1* [67]. Furthermore, *ABF3* expression can be downregulated at the post-transcriptional level by miR399f to release ABA-mediated growth arrest under stress [68]. ABFs/AREBs activity is also modulated by NACs at the protein level. Previously, ABF2/AREB1 and ABF4/AREB2 were found to bind with ANAC096 and cooperatively regulate the expression of stress-responsive genes [69]. Interestingly, the NAC072 interacts with ABF3 to enhance expression of *RD29A* and to decrease *RD29B* activity. Therefore, NAC072 exerts a differential type of action on ABF3 function in ABA signaling [70]. Recently, the activity of ABF2/AREB1 was shown to be likely enhanced by interaction with GA-related DELLA proteins. This could promote stomatal closure and drought tolerance. It was evidenced that the ABF2/AREB1 was also involved in ABA-GA crosstalk to ensure the efficient response to drought stress [71].

## 6. The Partially Overlapping Function of ABI5 and ABFs/AREBs in Seeds, Seedlings, and Vegetative Tissues

Although ABI5 is considered as the main ABA-dependent bZIP factor regulating stress responses in seeds and seedlings, ABFs/AREBs also seem to be active during Arabidopsis’ early developmental stages. The function of ABFs/AREBs during germination and early seedling development was already indicated by Kim et al. [72], Finkelstein et al. [73] and Sharma et al. [59]. They observed faster germination of *abf1*, *abf4/areb2* and *abf3* mutants under optimal growth conditions and better germination rate of *abf4/areb2* and *abf3* under ABA treatment [59,72]. Moreover, ABF3 acts redundantly with ABI5 in seeds and during post-germinative growth under abiotic stress [73]. It was also shown that ABF3 directly promotes *ABI5* expression in salt-treated seedlings [74]. Furthermore, ABF3 together with ABF1 regulate seed germination and post-germinative growth under heat stress. They directly activate expression of *CYSTEINE PROTEINASE INHIBITOR 5* (*CYS5*), a gene encoding an inhibitor of cysteine protease, leading to thermotolerant germination and growth of primary root [75] (Figure 1). *ABF1*, *ABF3* and *ABF4/AREB2* also regulate seed germination under salt and osmotic stress, downstream of *DE-ETIOLATED 1* (*DET1*), a negative regulator involved in light signaling pathway [76].

On the other side, ABI5 is active in vegetative tissues of Arabidopsis. Recently, You et al. [77] showed that ABI5 takes part in the adaptation of plant growth to a low CO_2_ level [78]. It was noted that ABI5 binds to the promoters of *SERINE:GLYOXYLATE AMINOTRANSFERASE 1* (*SGAT1*) and *GDC T-PROTEIN* (*GLDT1*) genes encoding enzymes associated with photorespiration process, and activates their expression [77] (Figure 1). Moreover, ABI5 is important for regulation of plant juvenile-to-adult transition. In response to ABA, MYB33, the main target of miR159, directly activates *ABI5* transcription. Next, ABI5 influences positively on *MIR156* expression, what in turn delays vegetative development under abiotic stress [79]. Furthermore, function of ABI5 and ABFs/AREBs can be overlapping in vegetative tissues. It has to be underlined that ABI5 can form heterodimers with ABFs/AREBs, which is the evidence of their synergistic role in ABA responses [8]. ABI5 was shown to inhibit photosynthesis and promote chlorophyll catabolism and leaf senescence [10,80,81] (Figure 1). However, ABFs/AREBs are also involved in regulating ABA-induced chlorophyll catabolism and leaf senescence in a similar way. ABF2/AREB1, ABF4/AREB2 and ABF3 directly activate expression of genes associated with chlorophyll catabolism: *STAY-GREEN 1/NON-YELLOWING 1* (*SGR1/NYE1*), *PHEOPHORBIDE A OXYGENASE* (*PAO*), *NON-YELLOW COLORING 1* (*NYC1*), and leaf senescence, *SENESCENCE-ASSOCIATED GENE 29* (*SAG29*) [82] (Figure 1). Besides Arabidopsis, ABI5 function was also observed during inhibition of chloroplast-related processes in potato (*Solanum tuberosum*). Similar to AtABI5, StABI5 negatively impacts photosynthesis and promotes chlorophyll catabolism and leaf senescence via positive regulation of *CHLOROPLAST VESICULATION* (*StCV*) and *StNYC1*, encoding proteins involved in chlorophyll degradation [83]. Moreover, in apple MdABI5 binds directly to promoters of *MdNYE1* and *MdNYC1* to promote chlorophyll catabolism and leaf senescence. MdABI5 transcriptional activity during regulation of leaf senescence is enhanced by its physical interaction with MdWRKY40 and MdbZIP44, but weakened by binding with MdBBX22 [84]. On the other side, dicot AtABF/AREB homologs, tomato (*Solanum lycopersicum*) SlAREB1 and sweetpotato (*Ipomoea batatas*) IbABF4 positively regulate photosynthesis efficiency in response to stress [85,86].

In vegetative tissues ABI5 can be also involved in response to biotic stress. *ABI5* expression is under epigenetic control of HOOKLESS1 (HLS1), which takes part in plant response to pathogens. It might imply that ABI5 is related to plant defense reaction against biotic stress [87]. Recently, it was shown that in tobacco (*Nicotiana benthamiana*), homolog of AtABI5, NbABI5 is able to directly repress the gene responsible for chloroplast electron transfer chain, *FERREDOXIN 1* (*NbFD1*), in rice stripe virus (RSV)-infected tobacco plants. *NbFD1* is also involved in callose deposition in plasmodesmata, which in turn protects plant against viral infection. Therefore, RSV-mediated activation of *NbABI5* can inhibit plant defense mechanism to virus [88]. It should be also underlined that tomato SlAREB1 participates in regulation of biotic stress-responsive genes such as *PATHOGENESIS-RELATED GENE 5* (*PR5*) or *CHITINASE3* (*CHI3*) [85]. Together, all these observations indicate that function of *ABI5* and *ABFs/AREBs* is not only restricted to seed germination and vegetative tissues, respectively. Furthermore, *ABI5* can function synergistically with *ABFs/AREBs* during ABA-dependent responses.

## 7. The Role of ABI5 and ABFs/AREBs in Flowering Regulation and Fruit Ripening

In Arabidopsis, ABFs/AREBs and ABI5 take part in ABA-dependent regulation of flowering timing. ABF4/AREB2 and ABF3 were shown to regulate flowering time and mediate the drought-escape mechanism. Both ABFs/AREBs interact with NUCLEAR FACTOR Y, SUBUNITs C3/4/9 (NF-YC3/4/9) in the promoter of *SUPPRESSOR OF OVEREXPRESSION OF CO 1* (*SOC1*), to promote its expression. *SOC1* encodes a MADS box transcription factor, an important regulator of flowering timing. ABA-induced expression of *SOC1* accelerates flowering under drought [89] (Figure 1). Moreover, Xiong et al. [90] observed that *ABI5* undergoes regulation at the post-transcriptional level during flowering transition. In the presence of ABA, splicing regulator, U2AF65b, increases efficiency of intron splicing from *ABI5* pre-mRNA, which promotes abundance of *ABI5* mature transcripts. This type of the *ABI5* expression control was observed in a shoot apex during ABA-mediated regulation of flowering transition [90].

Recently, some evidence emerged that ABI5 and ABFs/AREBs are also involved in fruit ripening and have an effect on fruit quality [91]. In Japanese plum (*Prunus salicina*), PsABI5 can directly activate the expression of ethylene biosynthesis gene, *ACC SYNTHASE1* (*PsACS1*) and thus, it takes part in fruit maturation [92]. It was also found that expression of *MdABI5* in apple is directly activated by KNOTTED1-LIKE HOMEOBOX 19 (MdKNOX19), ABA-responsive transcription factor involved in inhibition of fruit and seed development [93]. Additionally, apple MdAREB2 promotes accumulation of soluble sugars through regulation of expression of sugar transporter (*SUCROSE TRANSPORTER 2, MdSUT2* and *TONOPLAST MONOSACCHARIDE TRANSPORTER1, MdTMT1*) and α-/β-amylase (*MdAMY1, MdAMY3, MdBAM1* and *MdBAM3*) genes, which in turn affects fruit quality [94]. Moreover, MaABI5, a banana homolog of AtABI5, is associated with ABA-induced cold tolerance of banana (*Musa acuminata*) fruits [95].

## 8. Function of ABI5 and ABFs/AREBs Homologs in Monocots

In monocots, a function of ABI5 and ABFs/AREBs homologs is usually not restricted to the specific developmental stage as it is observed in Arabidopsis. Monocot homologs of AtABI5 can be active in seeds and vegetative tissues during ABA-dependent responses to abiotic stress [10,96,97,98,99]. It was observed that rice (*Oryza sativa*) OsABI5, OsABF1, OsABF2/ABI5-Like1 (OsABL1)/OsbZIP46, OsABF4/OsbZIP72, OsTRAB1, wheat (*Triticum aestivum*) TaABL1, ABRE BINDING PROTEIN 1 (TaABP1), TaAREB3, wABI5 and maize (*Zea mays*) ZmABP9, ZmABI5 are crucial components of abiotic stress responses in vegetative tissues because of ABA-dependent regulation of stress-responsive genes [97,98,99,100,101,102,103,104,105,106,107,108]. Moreover, barley (*Hordeum vulgare*) HvABI5, rice OsABF2/OsABL1/OsbZIP46, OsABF4/OsbZIP72 and maize ZmABP9 regulate ABA-dependent responses at seed germination stage [96,103,105,106,107]. Furthermore, another monocot ABI5 and ABFs/AREBs homologs, barley HvABF1, HvABF2, sorghum (*Sorghum bicolor*) SbABI5 and wheat TaABF1, are important for ABA-GA crosstalk during seed germination [109,110,111,112] (Table 1).

Recently, a set of new data has emerged regarding monocot AtABI5 and AtABFs/AREBs homologs. Ishibashi et al. [113] found that HvABI5, similarly to AtABI5, directly activates expression of *HvCAT2* and thus reduces H_2_O_2_ level in embryos and promotes seed dormancy. HvABI5 was also described as an ABA-dependent regulator of drought response in barley vegetative tissues via participation in stomatal closure, photosynthesis inhibition and flavonoid accumulation. It has to be underlined that HvABI5 enables drought adaptation by activation of stress-responsive genes and the induction of genes encoding core ABA pathway components [114]. Furthermore, Zhang et al. [120] found that *ZmABI5* is active in seeds and the encoded transcription factor directly promotes *GALACTINOL SYNTHASE2* (*ZmGOLS2*) gene expression associated with raffinose biosynthesis, which is essential for seed viability. Therefore, *HvABI5* and *ZmABI5* expression regulate ABA-dependent responses during seed germination and further developmental stages. However, in wheat, *TaABI5* was shown to be active only in seeds. It promotes *LEA* expression and maintains seeds in dormancy stage. Transgenic Arabidopsis lines overexpressing *TaABI5* were ABA-hypersensitive during seed germination [119,121]. Furthermore, as observed in Arabidopsis, TaJAZ1 is able to interact with TaABI5 and thus it inhibits ABA-dependent TaABI5 activity and promotes seed germination [40]. Similar activity of TaABI5 and AtABI5 indicates that TaABI5 can be a functional ortholog of AtABI5 in wheat. Piao et al. [116] found that OsABF4/OsbZIP72 is involved in chlorophyll catabolism and leaf senescence, such as it was observed for AtABI5 and AtABFs/AREBs. They showed that OsABF4/OsbZIP72 binds to promoters of genes responsible for chlorophyll catabolism, *OsSGR1* and *OsNYC1*, and activates their expression. Moreover, OsABI5 was shown to interact with KELCH-LIKE ECH-ASSOCIATED PROTEIN 1 (OsKEAP1) which in turn promotes OsABI5 proteasomal degradation in germinating seeds under non-stressed conditions [117]. To summarize, all these findings indicate that function of monocot ABA-dependent bZIPs under abiotic stress can be diversified. It might be more or less similar to their homologs in Arabidopsis (Table 1). However, it should be noted that monocot homologs of ABI5 and ABF/AREBs can be active at different developmental stages, whereas in dicot species their function is generally observed during specific timing. On the other side, some evidence has emerged about the role of dicot ABI5 and ABF/AREBs in ABA signaling during later developmental stages e.g., flowering, fruit development and during plant response to biotic stress. These findings have not yet been confirmed for ABI5 and ABF/AREBs in monocot species. It should be also pointed out that dicot ABI5 and ABF/AREBs positively regulate response to abiotic stress, whereas monocot ABI5 and ABF/AREB homologs can function as positive or negative regulators of stress response. Moreover, AtABI5 plays the important role in crosstalk between ABA and other phytohormonal pathways at germination stage. This function of ABI5 has not yet been fully revealed in monocot species. Therefore, further studies are needed to reveal their proper function under stress at different developmental stages.

## 9. Role of ABI5 and ABFs/AREBs in Feedback Regulation of Core ABA Pathway

**Figure 3:**
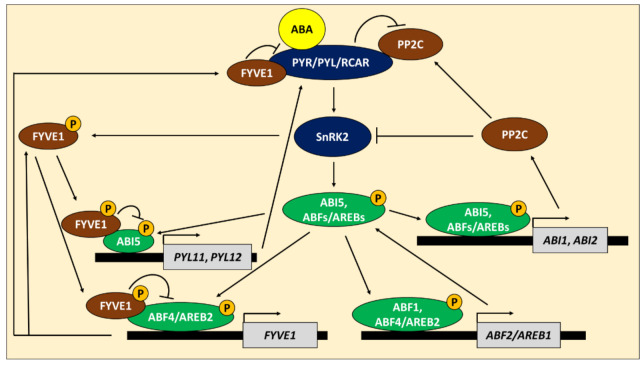
ABA INSENSITIVE 5 (ABI5) and ABRE BINDING FACTORs/ABRE-BINDING PROTEINs (ABFs/AREBs) function in feedback regulation of abscisic acid (ABA) pathway in Arabidopsis. In the presence of ABA, ABA receptors PYRABACTIN RESISTANCE PROTEINS/PYR-LIKE PROTEINS/REGULATORY COMPONENTS OF ABA RECEPTORs (PYR/PYL/RCARs) bind and inhibit phosphatases PHOSPHATASE 2Cs (PP2Cs), what in turn activates kinases, SNF1-RELATED PROTEIN KINASE 2s (SnRK2s). SnRK2s phosphorylate and activate ABI5 and ABFs/AREBs. Phosphatases PP2Cs, when not bound with ABA receptors PYR/PYL/RCAR, inhibit SnRK2s activity and thus ABI5 and ABFs/AREBs function. ABI5 is able to promote expression of *PYL11* and *PYL12* genes, which in turn strengthen ABA perception through PYR/PYL/RCAR receptors. ABF1 and ABF4/AREB2 trigger *ABF2/AREB1* expression. On the other side, ABI5 and ABFs/AREBs activate expression of *ABI1* and *ABI2*, genes encoding PP2Cs, which inhibits activity of SnRK2. Furthermore, ABF4/AREB2 promotes *FYVE DOMAIN PROTEIN REQUIRED FOR ENDOSOMAL SORTING 1* (*FYVE1*) expression. FYVE1 positively regulates degradation of PYR/PYL/RCARs and diminishes ABF4/AREB2 and ABI5 activity. Inhibition of ABF4/AREB2 and ABI5 activity by FYVE1 is possible after its phosphorylation carried out by SnRK2s. P—phosphate group.

Recently, growing evidence indicated the crucial role of ABI5 and ABFs/AREBs in the feedback regulation of the core ABA signaling and ABA biosynthesis. In Arabidopsis, Wang et al. [122] found that *ABI1* and *ABI2*, genes encoding group A PP2C phosphatases, negative regulators of ABA signaling, are directly activated by ABI5, ABF1, ABF2/AREB1, ABF4/AREB2 and ABF3 in the presence of ABA (Figure 3). Moreover, they noticed that *ABF2/AREB1* is a target gene of ABF1 and ABF4/AREB2. It shows that ABA-dependent bZIPs are part of the negative feedback loop in the ABA signaling. However, they can also strengthen their expression. Recently, ABI5 was described as a direct activator of genes encoding ABA receptors, *PYL11*, and *PYL12*, during seed germination under ABA (Figure 3). Furthermore, the pattern of eight other *PYL* genes’ expression is disturbed in *abi5* mutant [123]. Thus, ABI5 is also crucial for the reinforcement of ABA signaling. Moreover, the ABA pathway’s feedback regulation depends on intermediate regulators such as *FYVE DOMAIN PROTEIN REQUIRED FOR ENDOSOMAL SORTING 1* (*FREE1/FYVE1*). *FYVE1* encodes a protein localized in the peripheral membrane of late endosomal compartments and is involved in protein sorting. The expression of *FYVE1* is directly induced by ABF4/AREB2 [124] (Figure 3). However, FYVE1 protein interacts with all PYR/PYL/RCAR receptors and stimulates their degradation [124,125]. Moreover, SnRK2 kinases, SnRK2.2 and SnRK2.3, which mostly phosphorylate and activate ABI5 and ABF/AREBs, are also able to phosphorylate FYVE1 promoting its nuclear localization in response to ABA. In the nucleus, FYVE1 binds with ABI5 and ABF4/AREB2 and reduces their transactivation function [126]. Thus, ABF4/AREB2 is also involved in negative modulation of ABA-mediated stress responses through *FYVE1* regulation.

Similar interactions were also observed in other species. The overexpression of *StABI5* induces expression of *PYR/PYL/RCAR* and *SnRK2* genes in potato [83]. Furthermore, it was shown in barley that expression of *HvSnRK2.1*, *HvPP2C4* and key ABA biosynthesis gene, *CAROTENOID CLEAVAGE DIOXYGENASE 1* (*HvNCED1)*, is activated in *hvabi5* mutant, which presumably exhibits enhanced activity of HvABI5 protein. *HvSnRK2.1*, *HvPP2C4*, and *HvNCED1* are putative ABI5 direct target genes because of ABRE elements in their promoters [114]. It is noteworthy that rice ABA-related bZIP transcription factor, OsbZIP23, can promote directly the expression of *OsPP2C49* and *OsNCED4* [127]. Taken together, ABI5 and ABFs/AREBs act in the feedback regulation of ABA pathway, which is important for the fine-tuning of ABA-dependent responses, according to the surrounding environmental conditions.

## 10. Stress Tolerance of Transgenic Plants Overexpressing ABA-Dependent bZIPs

In Arabidopsis, transgenic lines overexpressing *ABFs/AREBs* showed increased tolerance to multiple abiotic stresses [57,72,128,129]. Moreover, rice, soybean (*Glycine max*) and cotton overexpressing Arabidopsis *ABF3, ABF2/AREB1* and *ABI5*, respectively, exhibited stress-tolerant phenotypes [130,131,132]. Therefore, ectopic expression of *ABFs/AREBs*, *ABI5* and their homologues can serve as a biotechnological tool for developing stress-tolerant cultivars. It was recently shown that potato overexpressing *ABF4/AREB2* was more tolerant to drought and salt stress. Plants with constitutive *ABF4/AREB2* expression accumulated more proline and exhibited decreased stomatal conductance and transpiration rate. Furthermore, tuber yield of transgenic lines was better than the wild-type, both, in the presence of optimal growth conditions and under stress [133]. In cotton, the overexpression of *AtABF3* and its cotton homolog, *GhABF2D*, resulted in drought tolerance related to the faster stomatal closure and reduced transpiration. However, transgenic lines also showed photosynthesis inhibition under drought [64]. Ectopic expression of *ABF3* also ensured increased tolerance of alfalfa (*Medicago sativa*) to drought, salt and oxidative stress. Drought tolerance of *ABF3* overexpression lines was caused by the reduction in transpiration rate, decreased ROS accumulation and higher chlorophyll content. On the other side, leaves of transgenic lines showed reduced size under optimal growth conditions [134]. The observed growth retardation could arise from constitutive expression of *ABFs/AREBs*. However, the application of stress-inducible or tissue-specific promoters can help to avoid this problem [64,72,129]. Na and Metzger [135] obtained transgenic plants of tomato and tobacco (*Nicotiana tabacum*) overexpressing *ABF4/AREB2* only in guard-cells of stomata. Transgenic lines showed reduced transpiration and ensured drought tolerance. Utilization of a guard-cell specific promoter reduced negative effect of *ABF4/AREB2* overexpression on plant development (Table 2).

Transgenic plants overexpressing homologs of *ABI5* and *ABFs/AREBs* from other species than Arabidopsis also showed better performance under stress [98,100,103,107,108,115]. Overexpression of wheat *TaAREB3* in Arabidopsis induced *LEA* expression and conferred freezing and drought tolerance [101]. Transfer of rapeseed (*Brassica napus*) gene, *BnaABF2*, to Arabidopsis ensured better tolerance to drought and salt due to the smaller stomatal aperture and induced expression of *LEAs*: *RD29B*, *RAB18* and *KIN2* [136]. Overexpression of maize *ZmABP9* in cotton resulted in tolerance to drought, salt and oxidative stresses. Transgenic lines showed a higher chlorophyll, proline and soluble sugars content, increased detoxifying enzymes activity, decreased ROS level and reduced stomatal aperture in response to stress. They also exhibited increased expression of stress-responsive genes under stress. Furthermore, seeds of transgenic lines germinated better under salt and osmotic stress [138]. Expression of *VvABF2* from grapevine (*Vitis vinifera*) in Arabidopsis caused increased activity of detoxifying enzymes, better ability to scavenge ROS and higher *LEA* expression. Together, it conferred tolerance to osmotic stress of transgenic lines [137]. Moreover, overexpression of sweet potato *IbABF4* in Arabidopsis and in sweet potato ensured tolerance to drought, salt and oxidative stresses, and lower ROS level, elevated ABA content, better performance of photosynthesis, and higher expression of *LEA* genes associated with stress. Additionally, Arabidopsis transgenic lines showed better germination rate under salt and osmotic stress [86] (Table 2).

Together, these data show that ABA-dependent bZIPs are a promising tool for developing cultivars with enhanced tolerance to abiotic stresses. However, the activity of ABA-dependent bZIPs can depend on a target species, type of applied stress and analyzed developmental stage. Moreover, overexpression of *ABFs/AREBs* and their homologs can also cause undesirable growth retardation under optimal conditions. Thus, laborious preliminary studies are needed before utilizing ABA-dependent bZIPs for obtaining stress-tolerant crops.

## 11. Concluding Remarks

ABA-dependent regulation of plant response to abiotic stress involves the activity of many different components. ABA-dependent bZIPs, ABI5 and ABFs/AREBs are a group of transcription factors that trigger plant adaptation to unfavorable stress conditions. Their activity is strictly controlled by multiple regulators at transcriptional and protein level to ensure the accurate response to surrounding environmental conditions. In Arabidopsis, ABI5 and ABFs/AREBs are mostly active in seeds and in vegetative tissues, respectively. However, very often their function is overlapping and together, in response to ABA, they regulate many processes including seed germination, chlorophyll catabolism and flowering time. In monocots, ABI5 and ABFs/AREBs homologs are involved in regulation of stress responses, however, their activity is usually observed during different developmental stages. Function of ABI5 and ABFs/AREBs is also crucial for the feedback regulation of core ABA pathway, which results in promotion or repression of ABA-dependent plant response to the stress. Finally, *ABI5*, *ABFs/AREBs* and their homologs can serve as candidates for developing transgenic plants with increased tolerance to abiotic stress. However, more studies are necessary to understand the precise function of numerous ABA-dependent bZIPs in regulation of plant responses to multiple abiotic stresses at different developmental stages.

## Figures and Tables

**Table 1:** Function of *ABI5* and *ABFs/AREBs* homologs in monocots.

Name	Source	GenBank ID/Ensembl Plants ID	Function	References
*HvABF1*	*Hordeum vulgare*	DQ786408/ HORVU3Hr1G084360	Inhibition of GA-induced expression of *Amy32b* in aleurone cells	[110]
*HvABF2*	*Hordeum vulgare*	DQ786409/ HORVU7Hr1G035500	Inhibition of GA-induced expression of *Amy32b* in aleurone cells	[110]
*HvABI5*	*Hordeum vulgare*	AY150676/ HORVU5Hr1G068230	ABA-dependent activation of *HVA1* and *HVA22* in aleurone cells	[96]
		AY150676/ HORVU5Hr1G068230	Direct activation of *HvCAT2* in dormant seeds	[113]
		HQ456390/ HORVU5Hr1G068230	ABA-dependent regulation of drought response including stomatal closure, flavonoid biosynthesis, photosynthesis inhibition and activation of stress-responsive and ABA pathway genes, regulation of seed germination under ABA	[114]
*OsABF1*	*Oryza sativa*	GQ904238/ Os01t0867300	Positive regulation of drought and salt stress responses through activation of stress-responsive genes	[104]
*OsABF2/* *OsABL1/* *OsbZIP46*	*Oryza sativa*	GU552783, XM_015785510/ Os06t0211200	Positive regulation of drought, salt and oxidative stress responses, ABA-dependent regulation of stress-responsive genes including *WRKYs*, participation in auxin responses, regulation of seed germination under ABA	[105,106,115]
*OsABF4/* *OsbZIP72*	*Oryza sativa*	AK065873, XM_015757064/ Os09g0456200	Positive regulation of drought response, regulation of seed germination under ABA, activation of chlorophyll catabolism genes: *OsSGR1* and *OsNYC1*	[103,116]
*OsABI5*	*Oryza sativa*	EF199631/ Os01t0859300	Negative regulation of drought and salt stress responses, involved in expression regulation of stress-responsive genes and in pollen maturation	[97]
			Interaction with OsKEAP1 at seed germination stage	[117]
*OsTRAB1*	*Oryza sativa*	AB023288/ Os08t0472000	ABA-dependent regulation of stress-responsive genes	[102]
*SbABI5*	*Sorghum bicolor*	XM_002454559/ SORBI_3004G309600	Activation of GA catabolism gene *SbGA2ox3* in embryos and promotion of seed dormancy	[111,118]
*TaABF1*	*Triticum aestivum*	AF519804/ TraesCS3A02G371800	Inhibition of GA-induced expression of *Amy32b* in aleurone cells via repression of *GAMyb* expression	[109,112]
*TaABP1*	*Triticum aestivum*	HQ166718/ TraesCS3B02G404300	Positive regulation of drought response	[108]
*TaABI5*	*Triticum aestivum*	AB238932/ TraesCS3D02G364900	Interaction with TaJAZ1, a negative regulator of JA signaling, at seed germination stage	[40]
			Regulation of seed dormancy and germination under ABA, activation of *LEA* expression	[119]
*TaABL1*	*Triticum aestivum*	BJ267580/ TraesCS6A02G333600	Positive regulation of drought, salt, freezing and oxidative stresses responses through promotion of chlorophyll accumulation, stomatal closure and stress-responsive genes expression, regulation of seed germination under ABA	[100]
*TaAREB3*	*Triticum aestivum*	-	Positive regulation of drought response through activation of stress-responsive genes	[101]
*wABI5*	*Triticum aestivum*	AB193553/ TraesCS5A02G237200	Positive regulation of drought, salt and freezing stresses responses through activation of stress-responsive genes	[98]
*ZmABP9*	*Zea mays*	GU237073/ Zm00001eb147240	Positive regulation of drought, salt, freezing and oxidative stresses responses through promotion of photosynthesis, stomatal closure, ROS scavenging and stress-responsive genes expression; regulation of seed germination under ABA; direct activation of *ZmCAT1*	[107]
*ZmABI5*	*Zea mays*	EU968937/ Zm00001d018178	Negative regulation of drought, salt, heat and cold stresses responses through promotion of chlorophyll catabolism and inhibition of detoxifying enzymes activity and proline accumulation, regulation of stress-responsive genes	[99]
			Activation of raffinose biosynthesis gene, *ZmGOLS2*, in seeds	[120]

**Table 2:** Abiotic stress tolerance of transgenic plants overexpressing *ABFs/AREBs* and their homologs, generated in recent years.

Gene	Source	Target Species	Effect	Reference
*ABF3*	*Arabidopsis thaliana*	*Medicago sativa*	Tolerance to drought, salt and oxidative stresses. Reduced transpiration rate, ROS content and higher chlorophyll content under stress. Smaller leaf area under optimal growth conditions.	[134]
*ABF3*	*Arabidopsis thaliana*	*Gossypium hirsutum*	Tolerance to drought. Reduced transpiration and photosynthetic rates. Slower growth and smaller leaves under optimal growth conditions.	[64]
*ABF4/AREB2*	*Arabidopsis thaliana*	*Solanum tuberosum*	Tolerance to drought and salt stress. Lower transpiration rate and higher proline content under stress. Improved tuber yield under optimal growth conditions and under stress.	[133]
*ABF4/AREB2*	*Arabidopsis thaliana*	*Nicotiana tabacum* and *Solanum lycopersicum*	Tolerance to drought. Reduced transpiration under stress. Mild growth reduction under optimal growth conditions.	[135]
*BnaABF2*	*Brassica napus*	*Arabidopsis thaliana*	Tolerance to drought and salt. Reduced stomatal aperture and water loss under stress. Induced expression of *LEA* genes under stress.	[136]
*GhABF2D*	*Gossypium hirsutum*	*Gossypium hirsutum*	Tolerance to drought. Reduced transpiration and photosynthetic rates. Slower growth under optimal growth conditions.	[64]
*IbABF4*	*Ipomoea batatas*	*Arabidopsis thaliana/* *Ipomoea batatas*	Tolerance to drought, salt and oxidative stresses. Higher photosynthetic efficiency, endogenous ABA content and lower ROS content under stress. Induced expression of *LEA* genes under stress. Better seed germination under salt and osmotic stress in Arabidopsis.	[86]
*TaAREB3*	*Triticum aestivum*	*Arabidopsis thaliana*	Tolerance to drought and freezing stresses. Lower ion leakage under stress. Induced expression of *LEA* genes under stress.	[101]
*VvABF2*	*Vitis vinifera*	*Arabidopsis thaliana*	Tolerance to osmotic stress. Higher activity of detoxifying enzymes activity and better ROS scavenging under stress. Induced expression of *LEA* genes under stress.	[137]
*ZmABP9*	*Zea mays*	*Gossypium hirsutum*	Tolerance to drought, salt and oxidative stresses. Higher chlorophyll, proline and soluble sugars content, higher activity of detoxifying enzymes, reduced stomatal aperture and ROS accumulation under stress, induced expression of stress-responsive genes. Better seed germination under salt and osmotic stress.	[138]

## Data Availability

Not applicable.
